# A reactivity-based [^18^F]FDG probe for *in vivo* formaldehyde imaging using positron emission tomography†
†Electronic supplementary information (ESI) available: Synthesis and characterization of probes, animal experiments, and supporting figures. See DOI: 10.1039/c6sc01503d


**DOI:** 10.1039/c6sc01503d

**Published:** 2016-05-25

**Authors:** Wei Liu, Charles Truillet, Robert R. Flavell, Thomas F. Brewer, Michael J. Evans, David M. Wilson, Christopher J. Chang

**Affiliations:** a Department of Chemistry , University of Berkeley , Berkeley , CA 94720 , USA . Email: chrischang@berkeley.edu; b Department of Radiology and Biomedical Imaging , University of California , San Francisco , California 94158 , USA . Email: David.M.Wilson@ucsf.edu; c Department of Molecular and Cell Biology , University of California , Berkeley , California 94720 , USA; d Howard Hughes Medical Institute , University of California , Berkeley , California 94720 , USA

## Abstract

Formaldehyde (FA) is a reactive carbonyl species (RCS) that plays a broad spectrum of roles in epigenetics, toxicology, and progression of diseases ranging from cancer to diabetes to neurodegeneration, motivating the development of translatable technologies for FA imaging. Here we report formaldehyde-caged-[^18^F]fluorodeoxyglucose-1 ([^18^F]FAC-FDG-1), an aza-Cope-based reactivity probe for *in vivo* FA imaging using positron emission tomography (PET). [^18^F]FAC-FDG-1 reacts selectively with FA over potentially competing analytes to generate [^18^F]FDG, allowing its FA-dependent uptake and retention in cell culture as well as in animal models. The relative uptake of [^18^F]FAC-FDG-1 was evaluated using FA-treated PC3 prostate cancer and U87-MG glioblastoma cells demonstrating a dose-dependent response to exogenously added FA. Moreover, [^18^F]FAC-FDG-1 is capable of FA detection *in vivo* using a PC3 tumor xenograft model. In addition to providing a unique tool for monitoring FA in living animals, these data establish a general approach for translatable detection of FA and other reactive biological analytes *in vivo* by exploiting the widely-available clinical [^18^F]FDG tracer as a masked aldehyde that can be caged by analyte-responsive triggers.

## Abstract 2

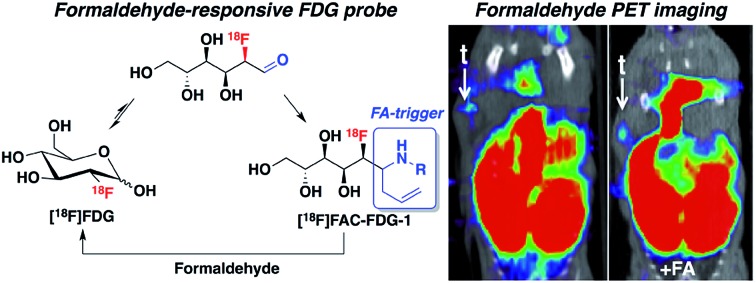
We present an aza-Cope-based reactivity probe for imaging formaldehyde *in vivo* using positron emission tomography.

## Introduction

Reaction-based chemical probes for selective and non-invasive molecular imaging of biologically important species have attracted significant attention. By utilizing biocompatible chemical transformations, a variety of small-molecule reagents have been developed to detect a diverse range of analytes in living systems.^1^–^5^ Among the many non-invasive molecular imaging techniques, fluorescence is currently the most well studied modality, particularly at the cellular level, owing to its high spatiotemporal resolution, high sensitivity, relative simplicity and the widespread use of confocal and other light microscopy. However, in part because of relatively poor tissue penetration, *in vivo* imaging with the fluorescence modality has had limited clinical translation compared to positron emission tomography (PET), which has been widely applied to oncology, neurology, cardiology and pharmacokinetic studies.^6^ As such, new chemical strategies for designing functional PET imaging agents for *in vivo* use are of interest, and in this context, reaction-based PET probes remain largely underdeveloped compared to radiolabeled ligands for receptors and other biomolecular targets.

**Scheme 1:**
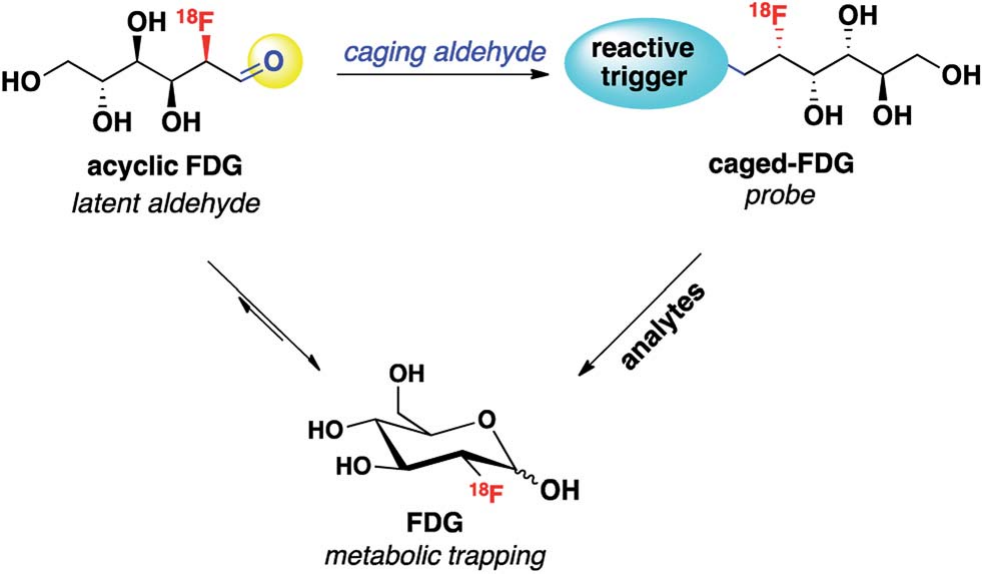
Caged ^18^F-FDG as a latent aldehyde for developing reaction-based PET probes.

One design strategy for bioanalyte sensing using PET relies on caging a clinically utilized PET tracer, as an analogy to reaction-based fluorescent probes that uncage useful dyes for light microscopy. In the presence of a specific bioanalyte, the caged species is degraded to the parent tracer, which can subsequently be trapped and accumulated in adjacent cells. We have recently employed this approach with success for PET-based monitoring of hydrogen peroxide^7^ and acidic pH.^8^ In view of the synthetic ease and wide availability of [^18^F]fluorodeoxyglucose (^18^F-FDG), the most commonly used PET tracer, we decided to pursue ^18^F-FDG as a general platform for developing reaction-based PET probes. In particular, we recognized that ^18^F-FDG could be thought of as a latent masked aldehyde and reasoned that the aldehyde group of this open-chain form of ^18^F-FDG could be converted to a reactive trigger through suitable chemical modification, which can selectively respond to the bioanalytes of interest and release parent ^18^F-FDG. Thus, the engineered ^18^F-FDG could be used as a reaction-based PET probe (Scheme 1).

To illustrate this concept with a representative example, we targeted the detection of formaldehyde (FA), a reactive carbonyl species (RCS) involved in a diverse array of processes related to human health and disease. Commonly used as a reagent for tissue preservation owing to its protein cross-linking ability,^9^ FA is a known carcinogen^10^ and has been associated with neurotoxicity and acute respiratory illness.^11^ FA is produced endogenously in the body by demethylation of histones, DNA/RNA, and various metabolites, mediated by enzymes including semicarbazidesensitive amine oxidase (SSAO),^12^,^13^ lysine-specific demethylase 1 (LSD1),^14^ and JmjC domain-containing histone demethylases (JHDM).^15^,^16^ More recent studies show that FA is essential for normal brain function, modulating DNA demethylation/methylation events that are critical for memory formation.^17^–^19^ FA homeostasis is maintained by the continuous action of FA-metabolizing enzymes, including mitochondrial ALDH2 and cytosolic ADH3,^20^,^21^ resulting in FA concentrations in healthy individuals ranging from 70 μM in blood to 200 μM in brain.^11^,^19^ However, elevation of formaldehyde-generating enzymes has been associated with many types of disease, including Alzheimer's disease,^22^,^23^ multiple sclerosis,^24^ heart disease,^25^ diabetes^26^ and different types of cancer.^27^–^30^ Indeed, FA levels reaching 700–1000 μM are observed in malignant tissues.^31^

These far-ranging roles of FA in healthy and diseased states motivate the development of new technologies for monitoring its spatial and temporal distributions in living systems. However, traditional methods for FA detection require sample processing and/or destruction including colorimetric assays,^32^ radiometry,^33^ HPLC^34^,^35^ and gas chromatography.^36^–^38^ Several fluorescent probes based on imine formation have been developed for detecting reactive aldehydes.^39^–^41^ As a first step to tracking FA in living samples, we and others have recently reported FA-responsive fluorescent probes based on aza-Cope reactivity that are selective for FA.^42^,^43^ Since then, some other fluorescent probes have been developed that can selectively image formaldehyde.^44^–^46^ With the goal of creating FA probes with potential for *in vivo* translation, we turned our attention to PET as a noninvasive nuclear medicine imaging modality. We now report the design, synthesis and application of formaldehyde-caged-[^18^F]fluorodeoxyglucose-1 ([^18^F]FAC-FDG-1), a unique PET probe for imaging FA in living animals that exploits the commonly-utilized clinical tracer [^18^F]FDG as a masked aldehyde. [^18^F]FAC-FDG-1 accumulates in cells upon cleavage of an FA-sensitive moiety and can be used to image FA levels within tumor xenografts in living animals.

## Result and discussion

### Design and synthesis of [^18^F]FAC-FDG-1 and [^18^F]Ctrl-FAC-FDG-1

**Fig. 1:**
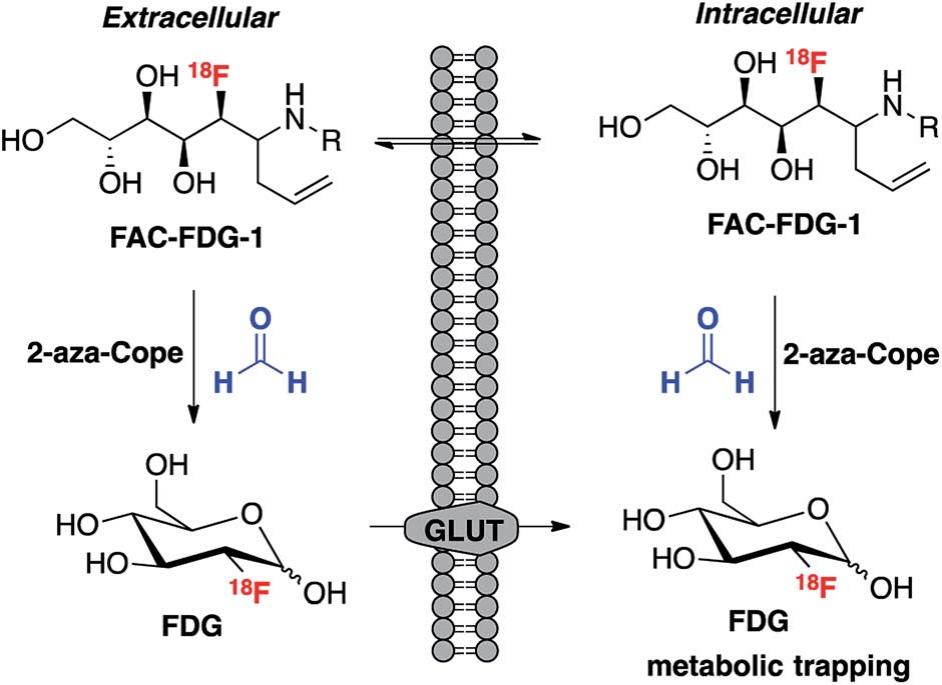
[^18^F]FAC-FDG-1, a PET tracer designed to exhibit FA-dependent cellular accumulation of [^18^F]FDG *via* aza-Cope uncaging of its masked aldehyde functionality.

We envisioned that the acyclic aldehyde form of [^18^F]FDG could be masked by a homoallylic amine, which would release the parent [^18^F]FDG tracer upon condensation with FA (Fig. 1). This glucose analogue is then transported into cells *via* the glucose transporter (GLUT) and subsequently phosphorylated by hexokinase (HK) resulting in its metabolic trapping.^47^,^48^ Indeed, the widespread availability of [^18^F]FDG has led to the its use in a variety of FDG derivatives bearing stable linkages.^49^–^53^ Based on these considerations we prepared [^18^F]FAC-FDG-1 (Fig. 1), noting that accumulation of intracellular [^18^F]FDG could result from either extracellular reaction-immolation of [^18^F]FAC-FDG-1 into [^18^F]FDG followed by GLUT transport or *via* passive diffusion of [^18^F]FAC-FDG-1 into cells and subsequent intracellular reaction with FA to generate [^18^F]FDG. In both cases, [^18^F]FDG would undergo phosphorylation by HK, resulting in trapped radiotracer and an accumulation in signal within cells with elevated levels of extracellular and/or intracellular FA.

**Scheme 2:**
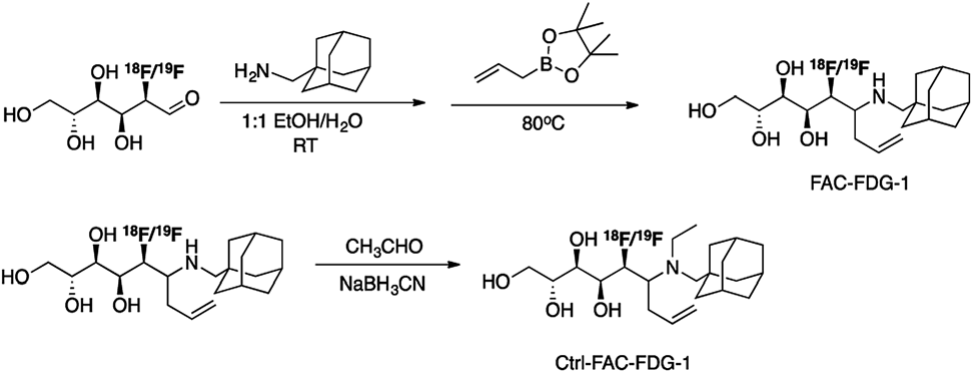
Synthesis of FAC-FDG-1 and Ctrl-FAC-FDG-1.

Scheme 2 outlines the synthesis of FAC-FDG-1 *via* aminoallylation of [^18^F/^19^F]FDG with adamantanemethyl amine and pinacol allylboronate.^54^ We reasoned that an adamantyl functionality would increase cell permeability.^55^ We also designed and synthesized the control probe Ctrl-FAC-FDG-1, which is identical to FAC-FDG-1 except for an ethyl group on the amine, rendering Ctrl-FAC-FDG-1 unable to condense with FA. Ctrl-FAC-FDG-1 was synthesized *via* reductive ethylation of FAC-FDG-1 with acetaldehyde. [^18^F]FAC-FDG-1 and [^18^F]Ctrl-FAC-FDG-1 were obtained in a 45 ± 13% (*n* = 6) and 14 ± 6% (*n* = 3) decay corrected radiochemical yields, respectively.

### Response and selectivity

**Fig. 2:**
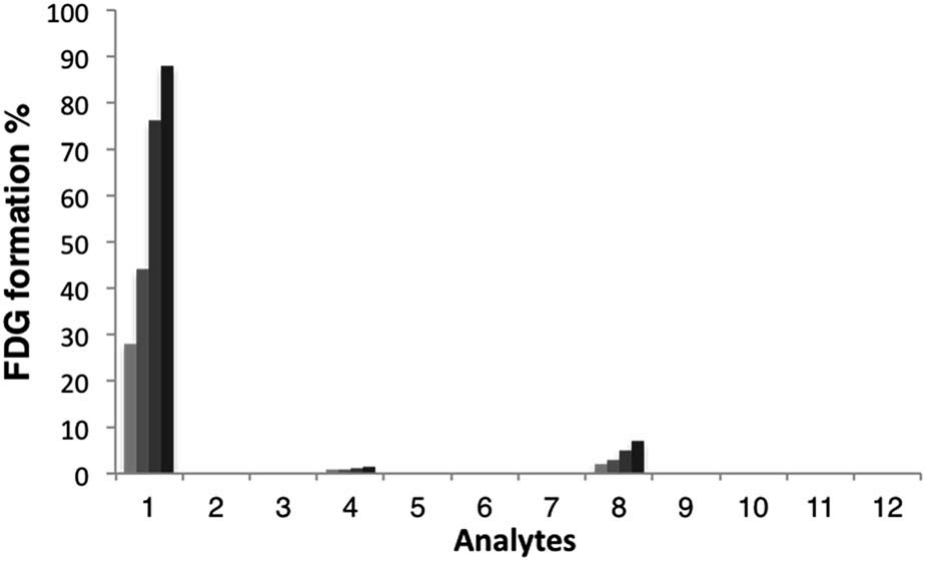
Relative conversions of [^18^F]FAC-FDG-1 to [^18^F]FDG upon treatment with biologically relevant RCS and related molecules. Bars represent formation of FDG at 30 (light grey), 60 (grey), 120 (dark grey) and 180 (black) min after addition. Data shown are for 1 mM of all species unless otherwise noted and were acquired in 20 mM PBS (pH 7.4) at 37 °C. Legend: (1) FA (2) PBS (3) [^18^F]Ctrl-FAC-FDG plus 1 mM FA (4) acetaldehyde; (5) glucose; (6) sodium pyruvate; (7) benzaldehyde; (8) methylglyoxal; (9) methylglyoxal (10 μM); (10) dehydroascorbic acid; (11) glucosone; (12) H_2_O_2_.

With these probes in hand, we then evaluated the reactivity of [^18^F]FAC-FDG-1 with FA and a variety of reactive carbonyl species (RCS) by monitoring its conversion to [^18^F]FDG using radio-HPLC (Fig. 2). In the presence of 1 mM FA under simulated physiological conditions, (20 mM, pH = 7.4 PBS), consumption of [^18^F]FAC-FDG-1 with concomitant formation of [^18^F]FDG was observed (Fig. S1†), leading to 43% and 76% conversions to product within 1 and 2 hours, respectively. In control experiments, no [^18^F]FDG formation was observed in the absence of FA or upon treatment of [^18^F]Ctrl-FAC-FDG-1 with FA under the same conditions. Moreover, [^18^F]FAC-FDG-1 shows high selectivity for FA over other potentially competing species, including acetaldehyde, glucose, sodium pyruvate, benzaldehyde, methylglyoxal, dehydroascorbic acid, glucosone, and hydrogen peroxide (Fig. 2). [^18^F]FAC-FDG-1 shows a small response to superphysiological level (1000 μM) of methylglyoxal, but is not responsive to 10 μM of this RCS, which is above its single-digit micromolar physiological range.^56^

### Cellular FA detection with [^18^F]FAC-FDG-1

**Fig. 3:**
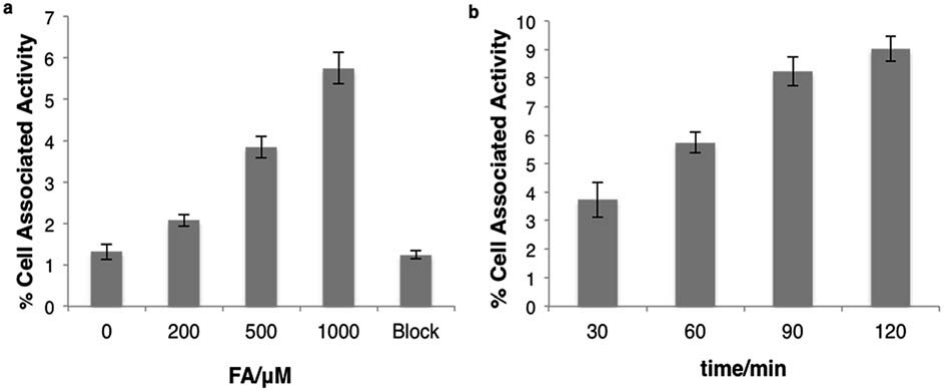
(a) Cellular uptake of [^18^F] in PC3 prostate cancer cells in the presence of [^18^F]FAC-FDG-1 upon treatment with 0, 200, 500, or 1000 μM FA or 1000 μM FA plus cytochalasin B (10 μg mL^–1^). (b) Time-dependent cellular uptake of [^18^F] in PC3 prostate cancer cells in the presence of [^18^F]FAC-FDG-1 upon treatment with 1000 μM FA.

We next tested whether [^18^F]FAC-FDG-1 could respond to changes in FA levels using PC3 prostate cancer and U87-MG glioblastoma cells, as these cell lines exhibit high FDG avidity. [^18^F]FAC-FDG-1 responses to added FA concentrations ranging from 0–1000 μM showed a FA dose-dependent (Fig. 3a) and time-dependent accumulation in cells (Fig. 3b), with a 4.4 fold increase in signal from 1.3 ± 0.2% cell associated activity at 0 μM FA to 5.7 ± 0.4% cell associated activity at 1000 μM FA at 1 h. Similarly, in U87-MG cancer cells, a 5.5-fold increase in signal was observed (Fig. S2 and S3†). The ctrl experiments showed that uptake of FDG in the same cell lines was not affected by varying FA concentrations (Fig. S5†). Moreover, [^18^F]Ctrl-FAC-FDG-1 did not exhibit a significant change in accumulation at 1 h ± 1 1000 μM FA (Fig. S4†). Also, at 1 h with 1000 μM FA, cell uptake of [^18^F]FAC-FDG-1 is effectively blocked by the addition of cytochalasin B,^57^ a known GLUT inhibitor, suggesting that [^18^F] accumulation occurs by GLUT-dependent transport. These data suggest that [^18^F]FAC-FDG-1 reacts with FA mainly *via* an extracellular process and the resulting [^18^F]FDG is transported intracellularly by GLUT and is then trapped by hexokinase.

### *In vivo* imaging of FA

**Fig. 4:**
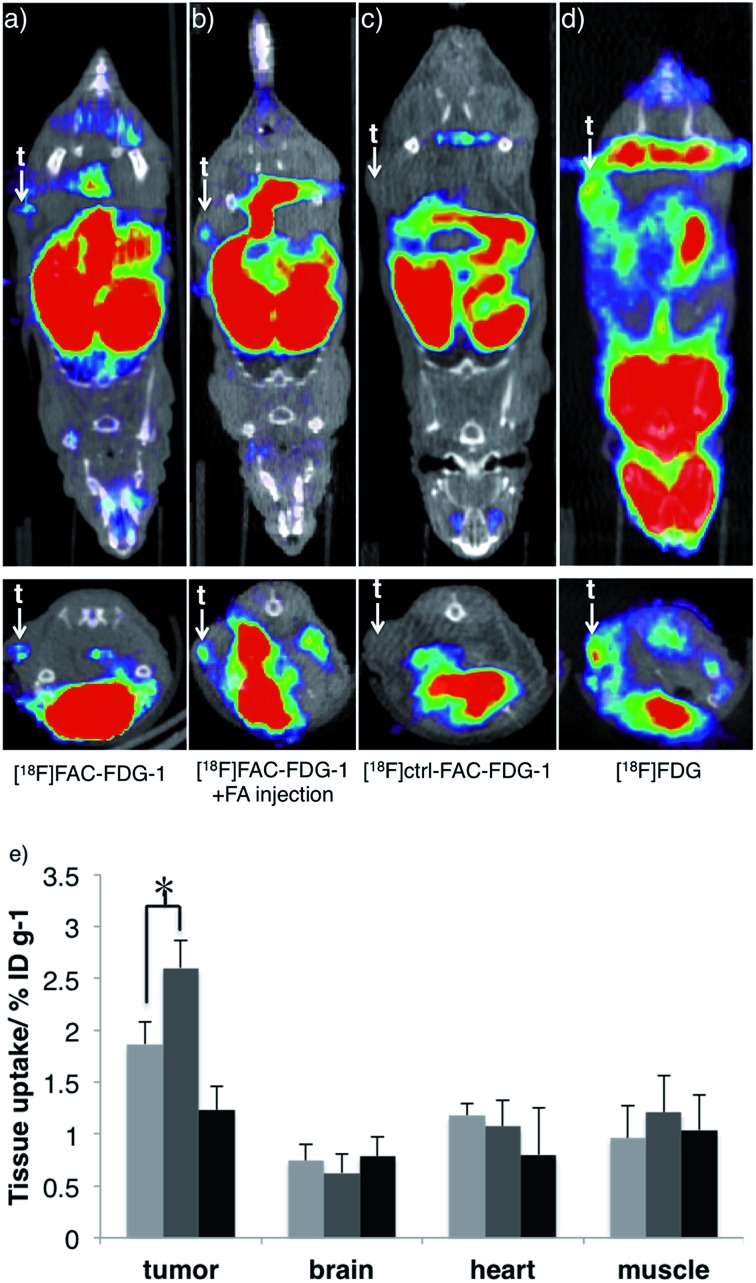
Representative [^18^F] positron emission tomography (PET) images of living mice bearing PC3-derived tumor xenografts administered with (a) [^18^F]FAC-FDG-1, (b) [^18^F]FAC-FDG-1 with intratumoral FA injection, (c) [^18^F]Ctrl-FAC-FDG-1, and (d) [^18^F]FDG. Top images show coronal view and bottom images show transverse view. t = tumor (e) relative [^18^F] uptake in various tissues as imaged with [^18^F]FAC-FDG-1 (light grey bars) [^18^F]FAC-FDG-1 plus 1 mM FA (grey bars), and [^18^F]Ctrl-FAC-FDG-1 (dark bars) *p < 0.05.

Finally, we evaluated the ability of [^18^F]FAC-FDG-1 to image changes in FA levels *in vivo* using a murine cancer model. Specifically, [^18^F] PET imaging was performed 7–8 weeks following implantation of PC3-derived xenograft tumors on the flanks of nu/nu mice. [^18^F]FAC-FDG-1 shows detectable uptake within the PC3-derived tumor as revealed by [^18^F] imaging in living mice (Fig. 4a), and the signal increases upon intratumoral injection of FA (Fig. 4b). As anticipated, the control probe [^18^F]Ctrl-FAC-FDG-1 does not exhibit significant uptake within tumor, with only hepatobiliary and renal clearance observed (Fig. 4c) and [^18^F]FDG providing a positive control (Fig. 4d and S7† for biodistribution). Biodistribution analysis of mice treated with [^18^F]FAC-FDG-1 and imaged establish that [^18^F] uptake in the tumor increased from 1.8 ± 0.26 ID% per g to 2.6 ± 0.24 ID% per g after the intratumoral injection of FA (*n* = 3, *p* < 0.05, data were analyzed using unpaired two-tailed Student's *t*-test; Fig. 4e and S6† for biodistribution in other organs). Taken together, these results demonstrate that [^18^F]FAC-FDG-1 is a new class of imaging tool for studying biological formaldehyde *in vivo*.

## Conclusions

To close, we have presented the design, synthesis, and cellular and *in vivo* properties of [^18^F]FAC-FDG-1, a unique reactivity-based PET probe for selective imaging of FA in living animals. [^18^F]FAC-FDG-1 reacts with FA *via* a 2-aza-Cope rearrangement to uncage the clinically-used PET tracer [^18^F]FDG in a FA-dependent manner, allowing for detection of changes of FA in living cells and animals most likely *via* an extracellular pathway. While we are encouraged by these proof-of-principle results, potential limitations may include the short ^18^F lifetime *vs.* FA uncaging as well as the short circulation time of the probe, and, therefore, future improvements will seek to improve probe kinetics by tuning the reactive trigger and optimize the pharmacokinetic properties of the probe. Current efforts are underway to apply [^18^F]FAC-FDG-1 and related reactivity-based imaging probes to various preclinical models, with particular interest in the epigenetic modifications seen in cancer and neurodegeneration.^55^,^58^,^59^ The use of aldehyde-caged [^18^F]FDG tracers provides a general synthetic platform for the potential design of a wide variety of responsive molecular imaging probes.

## Supplementary Material

Supplementary information

## References

[cit1] Chan J., Dodani S. C., Chang C. J. (2012). Nat. Chem..

[cit2] Chen X. Q., Tian X. Z., Shin I., Yoon J. (2011). Chem. Soc. Rev..

[cit3] Yang Y. M., Zhao Q., Feng W., Li F. Y. (2013). Chem. Rev..

[cit4] Cho D. G., Sessler J. L. (2009). Chem. Soc. Rev..

[cit5] Tang Y. H., Lee D. Y., Wang J. L., Li G. H., Yu J. H., Lin W. Y., Yoon J. Y. (2015). Chem. Soc. Rev..

[cit6] Ametamey S. M., Honer M., Schubiger P. A. (2008). Chem. Rev..

[cit7] Carroll V., Michel B. W., Blecha J., VanBrocklin H., Keshari K., Wilson D., Chang C. J. (2014). J. Am. Chem. Soc..

[cit8] Flavell R. R., Truillet C., Regan M. K., Ganguly T., Blecha J. E., Kurhanewicz J., VanBrocklin H. F., Keshari K. R., Chang C. J., Evans M. J., Wilson D. M. (2016). Bioconjugate Chem..

[cit9] Metz B., Kersten G. F. A., Hoogerhout P., Brugghe H. F., Timmermans H. A. M., de Jong A., Meiring H., ten Hove J., Hennink W. E., Crommelin D. J. A., Jiskoot W. (2004). J. Biol. Chem..

[cit10] Songur A., Ozen O. A., Sarsilmaz M. (2010). Rev. Environ. Contam. Toxicol..

[cit11] Tulpule K., Dringen R. (2013). J. Neurochem..

[cit12] Yu P. H., Wright S., Fan E. H., Lun Z. R., Gubisne-Harberle D. (2003). Biochim. Biophys. Acta, Proteins Proteomics.

[cit13] O'Sullivan J., Unzeta M., Healy J., O'Sullivan M. I., Davey G., Tipton K. F. (2004). Neurotoxicology.

[cit14] Shi Y. J., Lan F., Matson C., Mulligan P., Whetstine J. R., Cole P. A., Casero R. A., Shi Y. (2004). Cell.

[cit15] Cloos P. A. C., Christensen J., Agger K., Helin K. (2008). Genes Dev..

[cit16] Hou H. F., Yu H. T. (2010). Curr. Opin. Struct. Biol..

[cit17] Patra S. K., Patra A., Rizzi F., Ghosh T. C., Bettuzzi S. (2008). Cancer Metastasis Rev..

[cit18] Miller C. A., Campbell S. L., Sweatt J. D. (2008). Neurobiol. Learn. Mem..

[cit19] Tong Z. Q., Han C. S., Luo W. H., Wang X. H., Li H., Luo H. J., Zhou J. N., Qi J. S., He R. Q. (2013). Age.

[cit20] Galter D., Carmine A., Buervenich S., Duester G., Olson L. (2003). Eur. J. Biochem..

[cit21] Iborra F. J., Renaupiqueras J., Portoles M., Boleda M. D., Guerri C., Pares X. (1992). J. Histochem. Cytochem..

[cit22] Ferrer I., Lizcano J. M., Hernandez M., Unzeta M. (2002). Neurosci. Lett..

[cit23] Unzeta M., Sole M., Boada M., Hernandez M. (2007). J. Neural Transm..

[cit24] Airas L., Mikkola J., Vainio J. M., Elovaara I., Smith D. J. (2006). J. Neuroimmunol..

[cit25] Boomsma F., De Kam P. J., Tjeerdsma G., Van Den Meiracker A. H., Van Veldhuisen D. J. (2000). Eur. Heart J..

[cit26] Obata T. (2006). Life Sci..

[cit27] Kahl P., Gullotti L., Heukamp L. C., Wolf S., Friedrichs N., Vorreuther R., Solleder G., Bastian P. J., Ellinger J., Metzger E., Schule R., Buettner R. (2006). Cancer Res..

[cit28] Xiang Y., Zhu Z., Han G., Ye X., Xu B., Peng Z., Ma Y., Yu Y., Lin H., Chen A. P., Chen C. D. (2007). Proc. Natl. Acad. Sci. U. S. A..

[cit29] Lim S., Janzer A., Becker A., Zimmer A., Schule R., Buettner R., Kirfel J. (2010). Carcinogenesis.

[cit30] Hayami S., Yoshimatsu M., Veerakumarasivam A., Unoki M., Iwai Y., Tsunoda T., Field H. I., Kelly J. D., Neal D. E., Yamaue H., Ponder B. A. J., Nakamura Y., Hamamoto R. (2010). Mol. Cancer.

[cit31] Tong Z. Q., Luo W. H., Wang Y. Q., Yang F., Han Y., Li H., Luo H. J., Duan B., Xu T. L., Maoying Q. L., Tan H. Y., Wang J., Zhao H. M., Liu F. Y., Wan Y. (2010). PLoS One.

[cit32] Nash T. (1953). Biochem. J..

[cit33] Szarvas T., Szatloczky E., Volford J., Trezl L., Tyihak E., Rusznak I. (1986). J. Radioanal. Nucl. Chem..

[cit34] Soman A., Qiu Y., Li Q. C. (2008). J. Chromatogr. Sci..

[cit35] Su T., Wei Y., He R. Q. (2011). Prog. Biochem. Biophys..

[cit36] Janos E., Balla J., Tyihak E., Gaborjanyi R. (1980). J. Chromatogr. A.

[cit37] Ebeler S. E., Clifford A. J., Shibamoto T. (1997). J. Chromatogr. B: Biomed. Sci. Appl..

[cit38] Takeuchi A., Takigawa T., Abe M., Kawai T., Endo Y., Yasugi T., Endo G., Ogino K. (2007). Bull. Environ. Contam. Toxicol..

[cit39] Song H., Rajendiran S., Kim N., Jeong S. K., Koo E., Park G., Thangadurai T. D., Yoon S. (2012). Tetrahedron Lett..

[cit40] Zhou W., Dong H., Yan H., Shi C. X., Yu M. M., Wei L. H., Li Z. X. (2015). Sens. Actuators, B.

[cit41] Wang T., Douglass Jr E. F., Fitzgerald K. J., Spiegel D. A. (2013). J. Am. Chem. Soc..

[cit42] Brewer T. F., Chang C. J. (2015). J. Am. Chem. Soc..

[cit43] Roth A., Li H., Anorma C., Chan J. (2015). J. Am. Chem. Soc..

[cit44] Tang Y. H., Kong X. Q., Xu A., Dong B. L., Lin W. Y. (2016). Angew. Chem., Int. Ed..

[cit45] He L. W., Yang X. L., Liu Y., Kong X. Q., Lin W. Y. (2016). Chem. Commun..

[cit46] Dong B. L., Song X. Z., Tang Y. H., Lin W. Y. (2016). Sens. Actuators, B.

[cit47] Fletcher J. W., Djulbegovic B., Soares H. P., Siegel B. A., Lowe V. J., Lyman G. H., Coleman R. E., Wahl R., Paschold J. C., Avrill N., Einhorn L. H., Suh W. W., Samson'O D., Delbekell D., Gorman M., Shields A. F. (2008). J. Nucl. Med..

[cit48] Som P., Atkins H. L., Bandoypadhyay D., Fowler J. S., Macgregor R. R., Matsui K., Oster Z. H., Sacker D. F., Shiue C. Y., Turner H., Wan C. N., Wolf A. P., Zabinski S. V. (1980). J. Nucl. Med..

[cit49] Bormans G., Verbruggen A. (2001). J. Labelled Compd. Radiopharm..

[cit50] Patt M., Sorger D., Scheunemann M., Stocklin G. (2002). Appl. Radiat. Isot..

[cit51] Maschauer S., Pischetsrieder M., Kuwert T., Prante O. (2005). J. Labelled Compd. Radiopharm..

[cit52] Prante O., Einsiedel J., Haubner R., Gmeiner P., Wester H. J., Kuwert T., Maschauer S. (2007). Bioconjugate Chem..

[cit53] Namavari M., Cheng Z., Zhang R., De A., Levi J., Hoerner J. K., Yaghoubi S. S., Syud F. A., Gambhir S. S. (2009). Bioconjugate Chem..

[cit54] Sugiura M., Hirano K., Kobayashi S. (2004). J. Am. Chem. Soc..

[cit55] Wang C. N., Schroeder F. A., Wey H. Y., Borra R., Wagner F. F., Reis S., Kim S. W., Holson E. B., Haggarty S. J., Hooker J. M. (2014). J. Med. Chem..

[cit56] Rabbani N., Thornalley P. J. (2014). Nat. Protoc..

[cit57] Kletzien R. F., Springer A., Perdue J. F. (1972). J. Biol. Chem..

[cit58] Venneti S., Dunphy M. P., Zhang H. W., Pitter K. L., Zanzonico P., Campos C., Carlin S. D., La Rocca G., Lyashchenko S., Ploessl K., Rohle D., Omuro A. M., Cross J. R., Brennan C. W., Weber W. A., Holland E. C., Mellinghoff I. K., Kung H. F., Lewis J. S., Thompson C. B. (2015). Sci. Transl. Med..

[cit59] Zhu L., Ploessl K., Kung H. F. (2014). Chem. Soc. Rev..

