# Supplementary material for: A reactivity-based [18F]FDG probe for in vivo formaldehyde imaging using positron emission tomography
Source: Chem Sci. 2016 May 25;7(8):5503–7. doi: 10.1039/c6sc01503d (PMC6021783; doi:10.1039/c6sc01503d)
Supplement: Supplementary file 1 [file SC-007-C6SC01503D-s001.pdf]

Electronic Supplementary Material (ESI) for Chemical Science.
This journal is © The Royal Society of Chemistry 2016

Supplementary Information for

A Reactivity-Based [^{18}F]FDG Probe for *In Vivo* Formaldehyde Imaging Using Positron Emission Tomography

Wei Liu,[‡,†] Charles Truillet,[‡] Robert R. Flavell,[‡] Thomas F. Brewer,[†] Michael J. Evans,[‡] David M. Wilson,*[‡] Christopher J. Chang*[,†,§,⊥]

Departments of [†]Chemistry and [§]Molecular and Cell Biology and the [⊥]Howard Hughes Medical Institute, University of California, Berkeley, California 94720, United States

[‡]Department of Radiology and Biomedical Imaging, University of California, San Francisco, California 94158, United States

Table of Contents

GENERAL METHODS 2

CELL UPTAKE STUDIES 2

XENOGRAFT MICE 2

PET IMAGING 3

PROBE SYNTHESIS 3

MTT CELL PROLIFERATION ASSAY 7

BIODISTRIBUTION ASSAY 8

SUPPLEMENTARY FIGURES 9

General Methods

2-deoxy-2-F-fluorodeoxyglucose was purchased from Carbosynth LLC (San Diego, CA). All other compounds were purchased from Sigma-Aldrich. ^{1}H NMR and ^{13}C NMR spectra were collected in D_2O (Cambridge Isotope Laboratories, Cambridge, MA) at 25 °C on Bruker AVB-400 with ^{13}C operating frequencies of 101 MHz, at the College of Chemistry NMR Facility at the University of California, Berkeley. All chemical shifts are reported in the standard notation of parts per million relative to residual solvent peak at 4.80 ppm (D_2O) as an internal reference. Splitting patterns are indicated as follows: br, broad; s, singlet; d, doublet; t, triplet; m, multiplet; dd, doublet of doublets. Electrospray mass spectral analyses were carried out using a LC-MS (Agilent Technology 6130, Quadrupole LC/MS).

Cell Uptake Studies

For cell uptake experiments, 0.5 million PC3 or U87 cells in 0.5 mL PBS with given amount of FA were placed in 12-well plates. ~2 µCi of the PET probe in 2 µLPBS was added to each well. Following incubation at 37 °C, media was removed and the cells were washed twice with ice cold PBS. The cells were then lysed with 1ml of 1M NaOH and collected (cell associated fraction). The cell-associated fractions were counted in a gamma counter and expressed as a percentage of the total activity added per equal relative number of cells (as determined using a MTT Cell Proliferation Assay). Experiments were performed in triplicate.

Xenograft Mice

All mice were handled according to the Guide for the Care and Use of Laboratory Animals and accredited by the Association for Assessment and

Accreditation of Laboratory Animal Care. Mouse studies were carried out following the procedures approved by the Laboratory Animal Resource Center facilities of UCSF. For inoculation into nude mice, PC3 cells were washed with PBS, digested with trypsin, resuspended in DMEM containing fetal bovine serum, and pooled. After centrifugation, cells were resuspended in Matrigel (BD Biosciences Discovery Labware, Bedford, MA)-PBS (1:1) at a concentration of 5×10^6 cells per 100 μL. Cell/Matrigel mixture of 100 μL was injected subcutaneously into 6-week-old male *nu/nu* mice (Charles River, Wilmington, Massachusetts) on the right flank surface. Tumor volumes and body weights were monitored the day of the imaging experiments. Mice were sacrificed after the imaging and tumors and several essential organs were removed for biodistribution analysis.

PET Imaging

All animals were fasted 4-5 hours before PET imaging exams. Under isofluorane anesthesia, a tail vein catheter was placed. Approximately 200 μCi of FDG, FAC-FDG-1, or Ctrl-FAC-FDG-1 was injected via the tail vein catheter. The animals were then placed in original cage. At one hour post-injection, the animals were transferred to a Siemens Inveon micro PET-CT system (Siemens, Erlangen, Germany), and imaged using a single static time frame of 10 minutes. A micro-CT scan was then obtained for attenuation correction and anatomical coregistration. No adverse events were observed during or after injection of any compound. Anesthesia was maintained during imaging using isofluorane. All spectra were viewed using open source Amide software (amide.sourceforge.net).

Probe Synthesis

^{18}F Probe Synthesis (FAC-FDG-1)

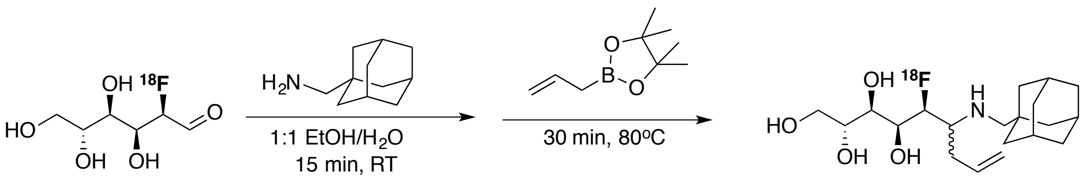

FAC-FDG-1

[18]F]FDG (~15 mCi) was dried by evaporation at 110 °C under vacuum and nitrogen stream. To the dried ^{18}F]FDG was added a solution of 25 μL adamantanemethylamine (0.14 mmol) in 400 μL 1:1 EtOH/H_2O. The resulting solution was stirred at room temperature for 15 min. 25 μL pinacol allylboronate (0.14 mmol) was then added and the reaction mixture was heated at 80 °C for 30 min. The resulting mixture was then purified using semi-preparative HPLC on a gradient of 0-100% B over 40 minutes, where A = water with 0.1% formic acid, and B = acetonitrile with 0.1% formic acid. The collected solution was dried by evaporation at 80 °C under vacuum and nitrogen stream (~3 mCi final product).

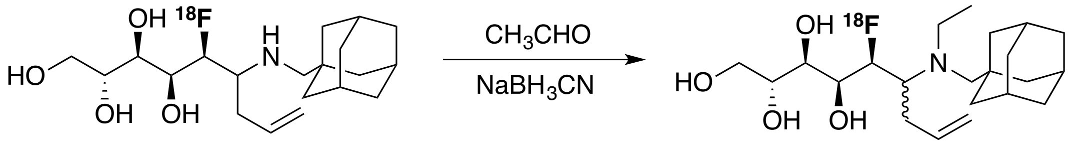

Ctrl-FAC-FDG-1

Control ^{18}F Probe Synthesis (ctrl-FAC-FDG-1)

[^{18}F]FAC-FAG-1 was redissolved in 200 μL ethanol. To the corresponding solution was added 10 μL acetaldehyde and 2 mg sodium cyanoborohydride. The reaction mixture was heated at 50 °C for 1h and was then purified using semi-preparative HPLC on a gradient of 0-100% B over 40 minutes, where A = water with 0.1% formic acid, and B = acetonitrile with 0.1% formic acid. The collected solution was dried by evaporation at 80 °C under vacuum and nitrogen stream.

^{19}F FAC-FDG-1 Synthesis

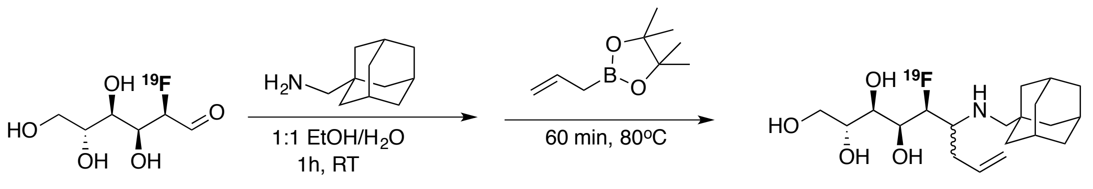

FAC-FDG-1

30 mg (0.16 mmol) FDG and 28 mg adamantanemethylamine (0.17 mmol, 1.06 eq.) was dissolved in 400 μL 1:1 EtOH/H_2O. The solution was stirred at room temperature for 1h. 29 mg pinacol allylboronate (0.17 mmol, 1.06 eq.) was then added and the reaction mixture was heated at 80°C for 60 min. The resulting mixture was then purified on Waters mass directed UHPLC system with 80% A and 20% B, where A = water with 0.05% formic acid, and B = acetonitrile with 0.05% formic acid. Pure fractions were combined and lyophilized to yield the title product as a white solid. 25 mg (36% yield). Isolated as a mixture of diastereomers. ^{1}H NMR (400 MHz, D_2O): δ 8.44 (s, 1H), 5.80 (m, 1H), 1.99 (m, 2H), 4.31 – 3.40 (m, 6H), 3.08 – 2.46 (m, 4H), 1.86 – 1.52 (m, 16 H). note: hydroxyl protons not observed. ^{13}C NMR (101 MHz, D_2O), 131.41, 120.62, 91.68, 70.61, 68.47, 62.60, 57.53, 50.69, 38.95, 38.76, 35.89, 37.79, 31.96, 27.62, 23.73. HRESI/MS m/z $C_{20}H_{35}FNO_4$ $(m+H)^+$ found 372.2561, cal'd 372.2550.

^{19}F-ctrl-FAC-FDG-1 Synthesis

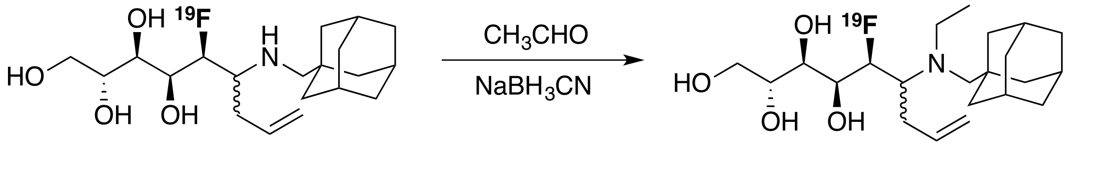

Ctrl-FAC-FDG-1

5 mg FAC-FDG-1 (0.016 mmol) was dissolved in 200 μL ethanol. To the corresponding solution was added 30 μL acetaldehyde (0.53 mmol) and 5 mg sodium cyanoborohydride (0.08 mmol). The solution was stirred at room temperature for 2h. The resulting mixture was then purified on Waters mass directed UHPLC system with 85% A and 15% B, where A = water with 0.05% formic acid, B = acetonitrile with 0.05% formic acid. Pure fractions were

combined and lyophilized to yield the title product as a white solid. 1.1 mg (19% yield). Isolated as a mixture of diastereomers. ^{1}H NMR (400 MHz, D_2O): δ 5.94 (m, 1H), 5.32 (m, 2H), 4.25 (m, 1H), 3.80 (m, 3H), 3.63 (m, 2H), 3.44 (m, 1H), 3.19 (m, 1H), 3.01 – 2.59 (m, 3H), 2.05 (s, 1H), 2.02 (s, 3H), 1.80 – 1.60 (m, 13 H), 1.35 (m, 3H). note: hydroxyl protons not observed. ^{13}C NMR δ 137.63, 114.56, 99.94, 71.65, 69.50, 69.38, 65.82, 64.56, 64.39, 63.54, 41.23, 36.82, 35.74, 28.62, 28.01, 13.69; HRESI/MS m/z $C_{20}H_{39}FNO_4$ $(m+H)^+$ found 400.2870, cal'd 400.2863

Selectivity Tests

990 μL of a 50 μCi [^{18}F]FAC-FDG-1 solution in PBS was incubated with corresponding analytes at 37°C. At indicated time, 100 μL of the solution was analyzed by radio-HPLC.

FA: 10 μL of 100 mM stock solution of FA in PBS buffer (freshly prepared by diluting 7.52 μL commercial 37 wt. % FA solution to 1mL with PBS) was added to 990 μL of [^{18}F]FAC-FDG-1 in PBS.

Acetaldehyde: 10 μL of 100 mM stock solution of acetaldehyde in PBS buffer (freshly prepared by diluting 5.6 μL neat acetaldehyde to 1mL with Milli-Q water) was added to 990 μL of [^{18}F]FAC-FDG-1 in PBS.

Glucose: 10 μL of 100 mM stock solution of glucose in PBS buffer (freshly prepared by dissolving 180 mg of glucose in 1 mL PBS) was added to 990 μLof [^{18}F]FAC-FDG-1 in PBS.

Methylglyoxal (1mM): 10 μL of 100 mM stock solution of methylglyoxal in PBS buffer (freshly prepared by diluting 15.4 μL of neat methylglyoxal to 1 mL with PBS) was added to 990 μL [^{18}F]FAC-FDG-1 in PBS.

Methylglyoxal (10 μM): 10 μL of 1 mM stock solution of methylglyoxal in PBS buffer (freshly prepared by diluting 10 μLof 100 mM stock solution of methylglyoxal in PBS to 1 mL with PBS) was added to 990 μL [^{18}F]FAC-FDG-1 in PBS.

H_2O_2: 10 μL of 100 mM stock solution of H_2O_2 in PBS (freshly prepared by diluting 10.2 μL of commercial 9.8 M H_2O_2 to 1 mL with PBS) was added to 990 μL of [^{18}F]FAC-FDG-1 in PBS.

Benzaldehyde: 1μL of 100 mM stock solution of benzaldehyde in CH_3CN (freshly prepared by dissolving 10.2μL benzaldehyde in 1mL CH_3CN) was added to 999 μL of [^{18}F]FAC-FDG-1 in PBS.

Sodium pyruvate: 10 μL of 100 mM stock solution of sodium pyruvate in PBS buffer (freshly prepared by dissolving 11 mg of sodium pyruvate in 1 mL Milli-Q water) was added to 990 μL of [^{18}F]FAC-FDG-1 in PBS.

Dehydroascorbate: 10 μL of 100 mM stock solution of dehydroascorbic acid in Milli-Q water (freshly prepared by dissolving 3.5 mg of dehydroascorbic acid in 200 μL Milli-Q water) was added to 990 μL of [^{18}F]FAC-FDG-1 in PBS.

Glucosone: 10 μL of 100 mM stock solution of glucosone in PBS buffer (freshly prepared by dissolving 3.6 mg of gluocosone in 200 μL Milli-Q water) was added to 990 μL of a [^{18}F]FAC-FDG-1 in PBS.

MTT Cell Proliferation Assay

The viability of PC3 and U87 cells was assessed using a 3-[4,5-dimethylthiazol-2-yl]-2,5-diphenyl tetrazolium bromide (MTT) dye reduction assay (Sigma-Aldrich). The cells were seeded in a 96-well plate at a density of 1 × 10^4 cells/well, cultured for 24 hours, then treated with different

concentration of formaldehyde. At the end of the treatment, MTT, 50 µg/10 µL, was added, and the cells were incubated for another 3 hours. MTT stock solution (5 mg/ml) is added to each culture being assayed to equal one-tenth the original culture volume and incubated for 3 to 4 hr. At the end of the incubation period the resultant formazan crystals were dissolved in dimethyl sulfoxide (100 µl) and the absorbance intensity measured by a microplate reader (TECAN Infinite M200) at 570 nm with a reference wavelength of 620 nm; all measurements were performed six times. The cell viability is typically higher than 85% in the presence of 1mM formaldehyde and the cell-associated activity is corrected by the cell viability.

Biodistribution Assay

After tumors grew to approximately 200-300 mm^3, a biodistribution assay was performed. All animals were fasted starting the 5 hours before PET biodistribution to minimize muscle uptake and optimize tumor to background signal. Under isofluorane anesthesia, a tail vein catheter was placed. ~ 200 µCi of the probe was injected via the tail vein catheter. The animals were then placed on in the original cage. At one hour post injection, the animals were sacrificed under isofluorane anesthesia. The indicated organs and blood were harvested and weighed, and the activity counted using a gamma counter. Calibration with known amounts of ^{18}F was performed to determine the amount of activity in each organ. The activity in each organ was decay-corrected, and the percent injected dose per gram of tissue was calculated and reported.

Supplementary Figures

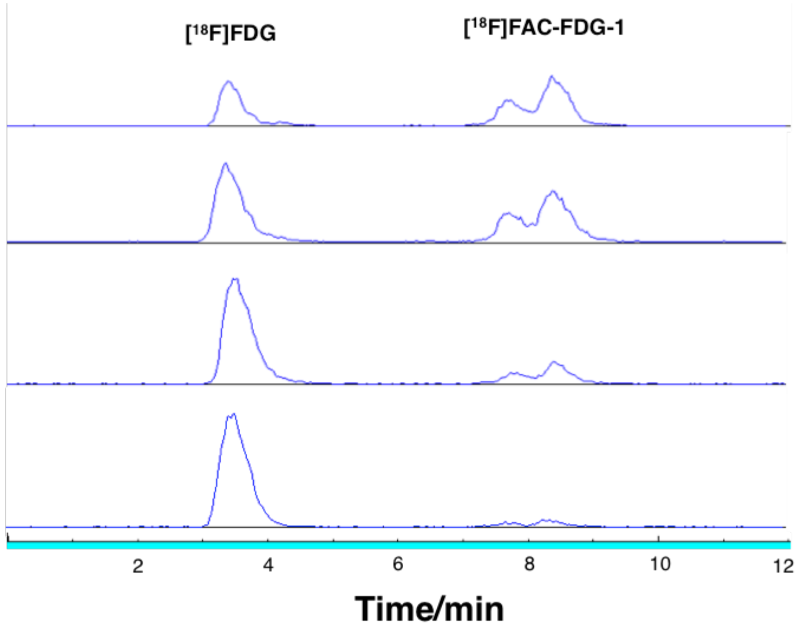

Figure S1. Radio-HPLC traces of FDG generation from FAC-FDG-1 in the presence of 1000 μM FA at 30 min (top trace), 60, 120, and 180 min (bottom trace). FAC-FDG-1 shows two peaks because of the formation of diastereomers.

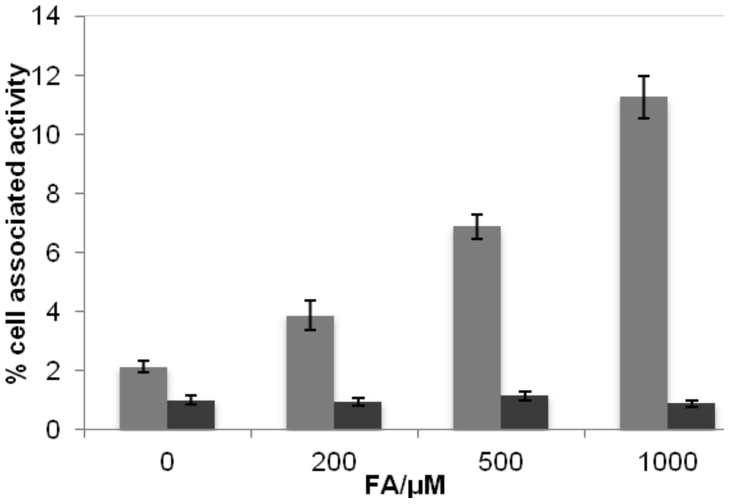

Figure S2. Cellular uptake of FAC-FDG-1 (light grey) and Ctrl-FAC-FDG-1 (dark grey) in U87 cells in the presence of 0, 200, 500, and 1000 μM FA.

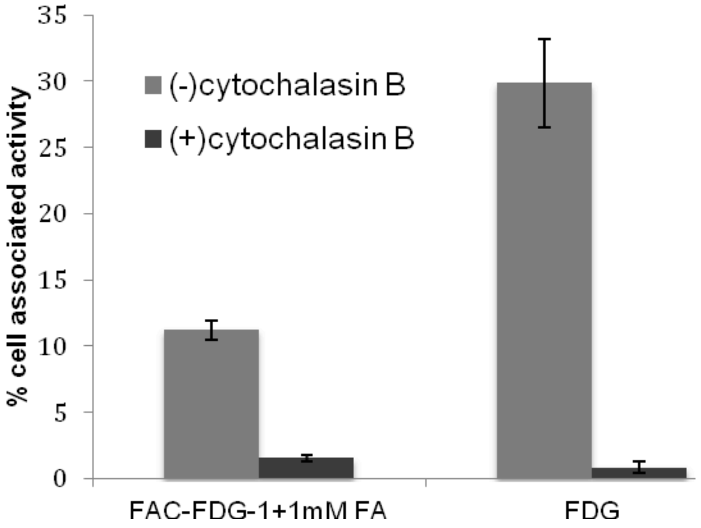

Figure S3. Effect of cytochalasin B on the cellular uptake of [^{18}F]FAC-FDG-1and [^{18}F]FDG in U87 cells.

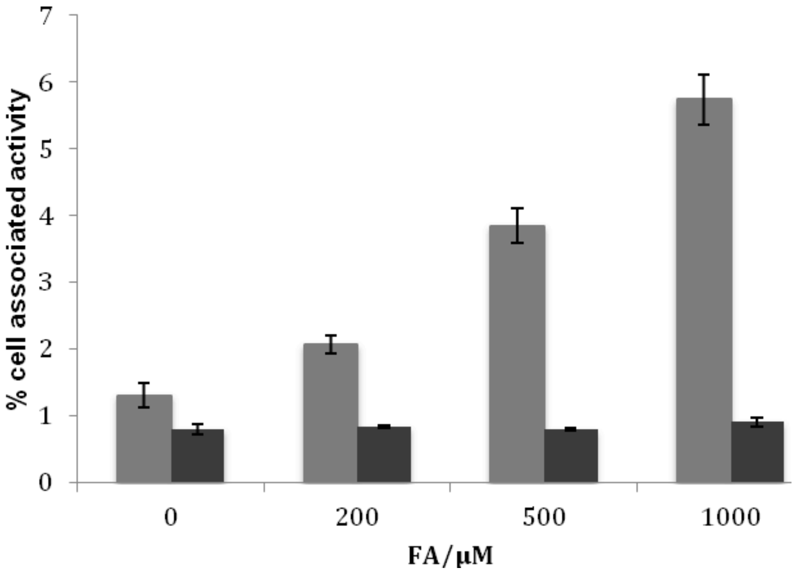

Figure S4. Cellular uptake of FAC-FDG-1 (light grey) and Ctrl-FAC-FDG-1 (dark grey) in the PC3 cell line in the presence of 0, 200, 500 and 1000 µM FA.

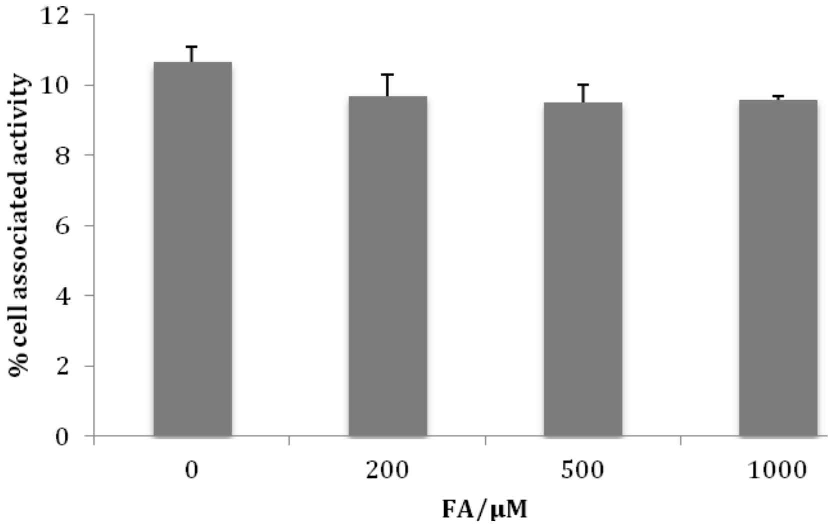

Figure S5. Cellular uptake of FDG in PC3 cell line in the presence of 0, 200, 500 and 1000 µM FA.

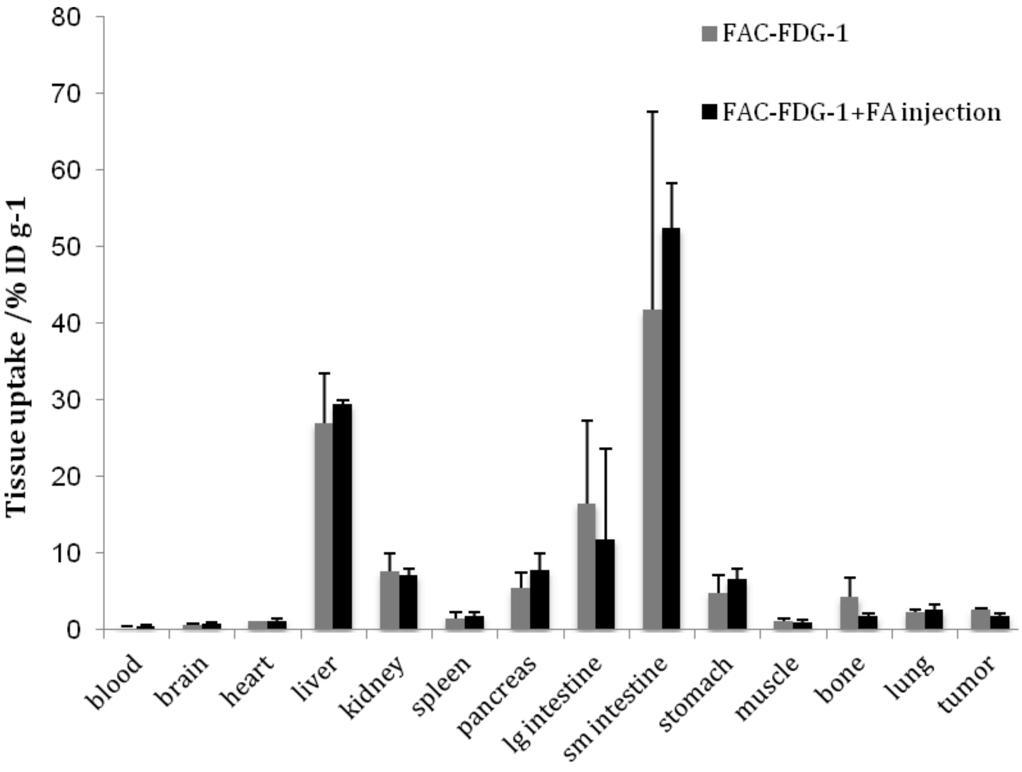

Figure S6. Biodistribution of FAC-FDG-1 in all the tissues of PC3 xenograft mice.

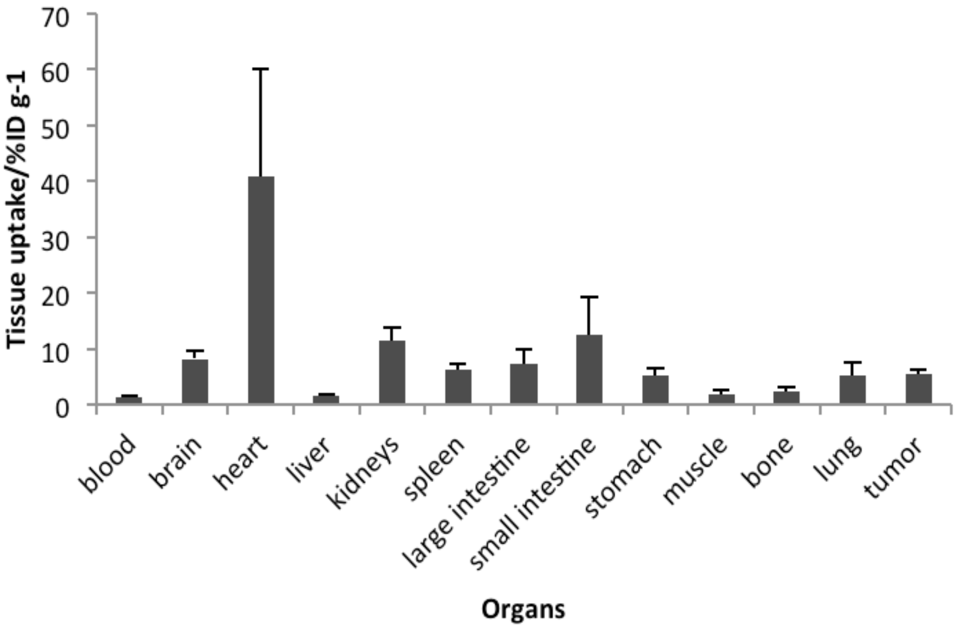

Figure S7. Biodistribution of FDG in PC3 xenograft mice.

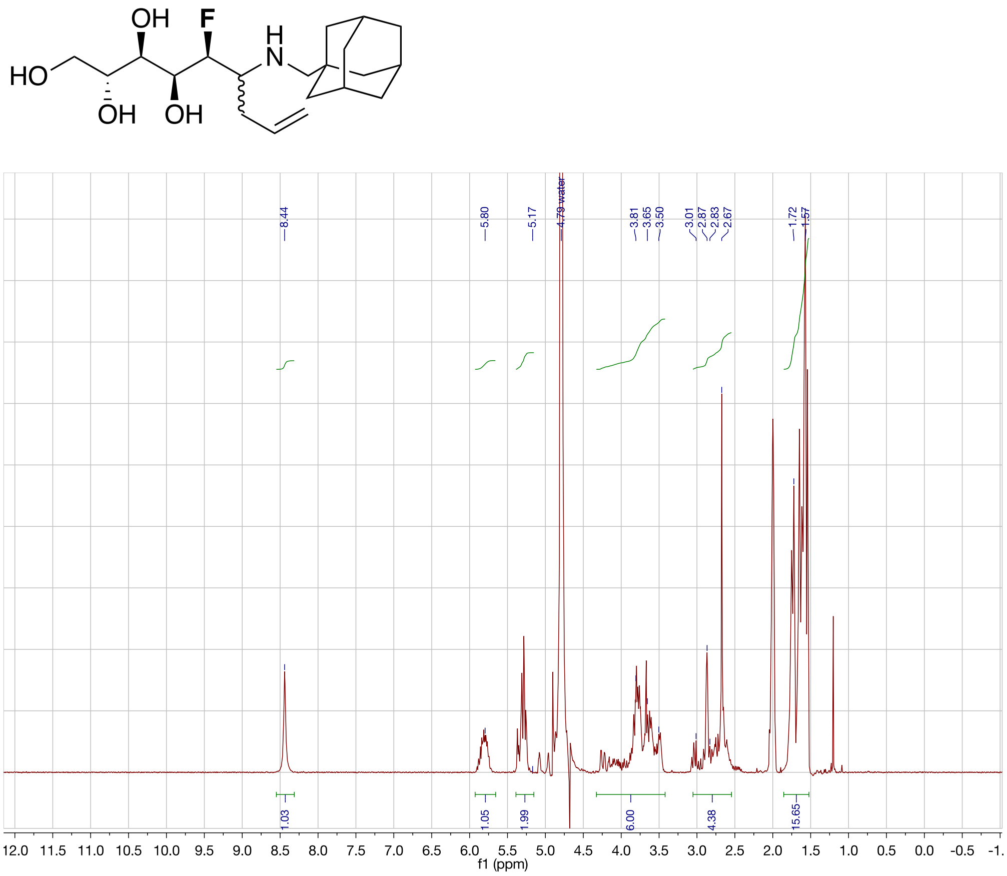

Figure S8. ^{1}H NMR spectra of [^{19}F]FAC-FDG-1

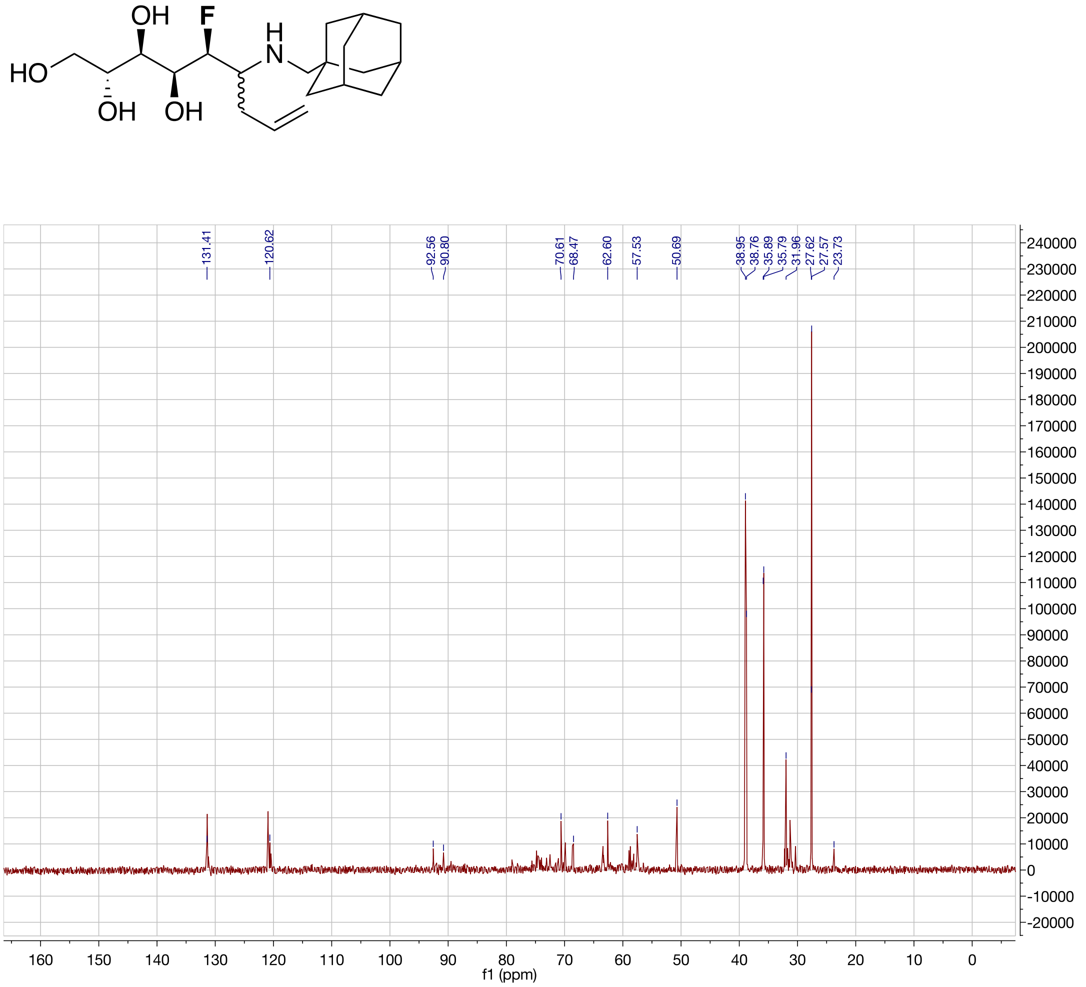

Figure S9. ^{13}C NMR spectra of [^{19}F]FAC-FDG-1.

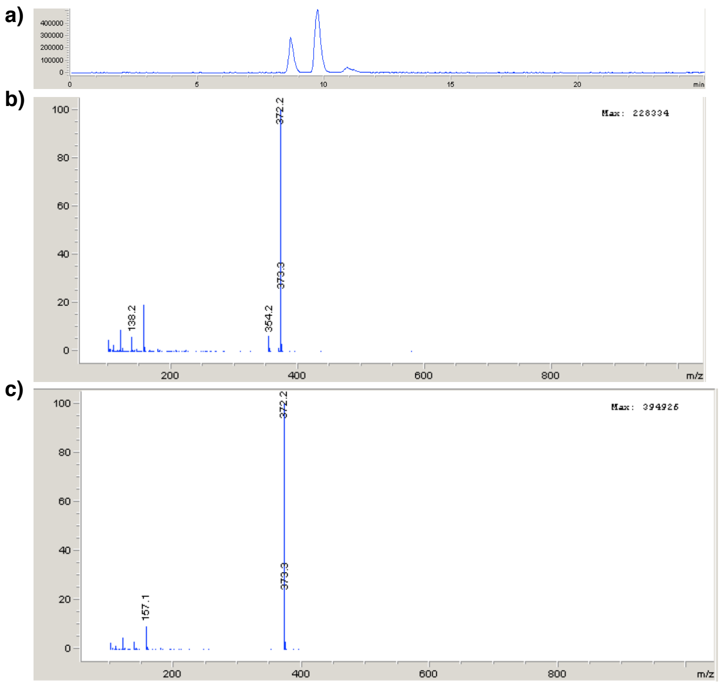

Figure S10. HPLC-MS analysis of [^{19}F]FAC-FDG-1 a) LC-MS trace of [^{19}F]FAC-FDG-1 showing the presence of two diastereomers b) ESI-MS of left peak c) ESI-MS of right peak

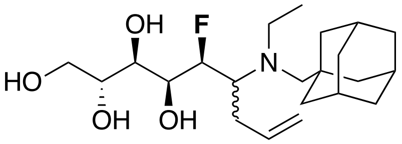

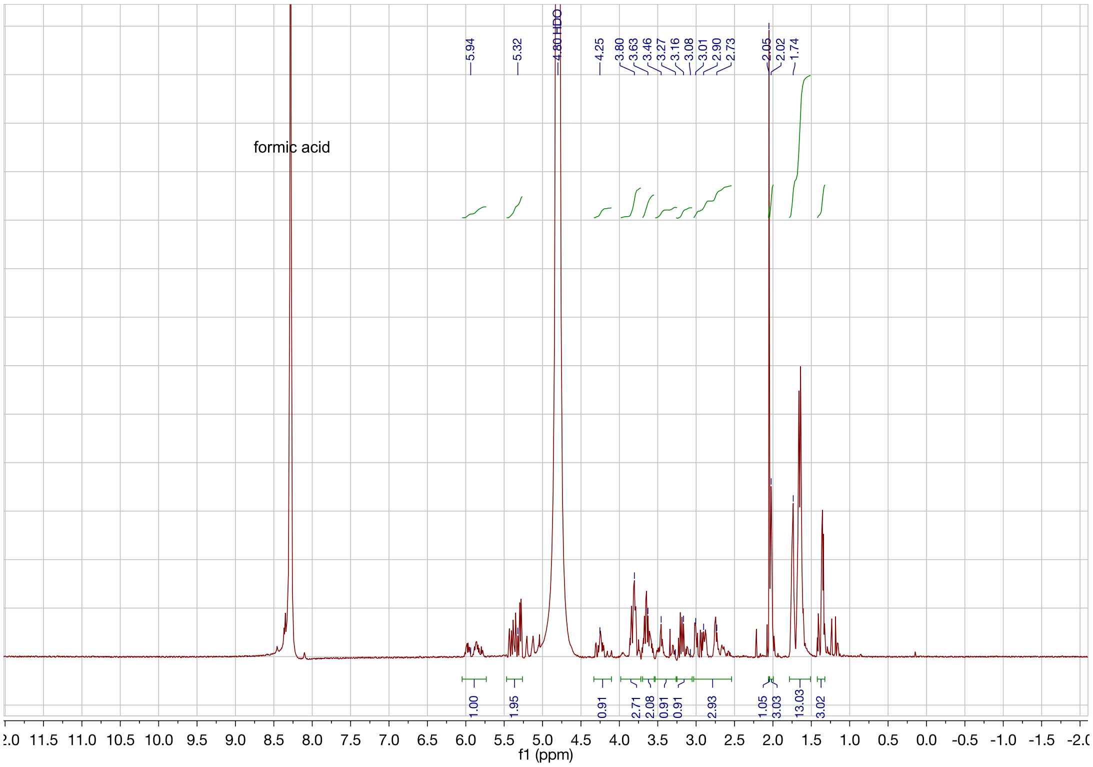

Figure S11. ^{1}H NMR spectra of [^{19}F]Ctrl-FAC-FDG-1.

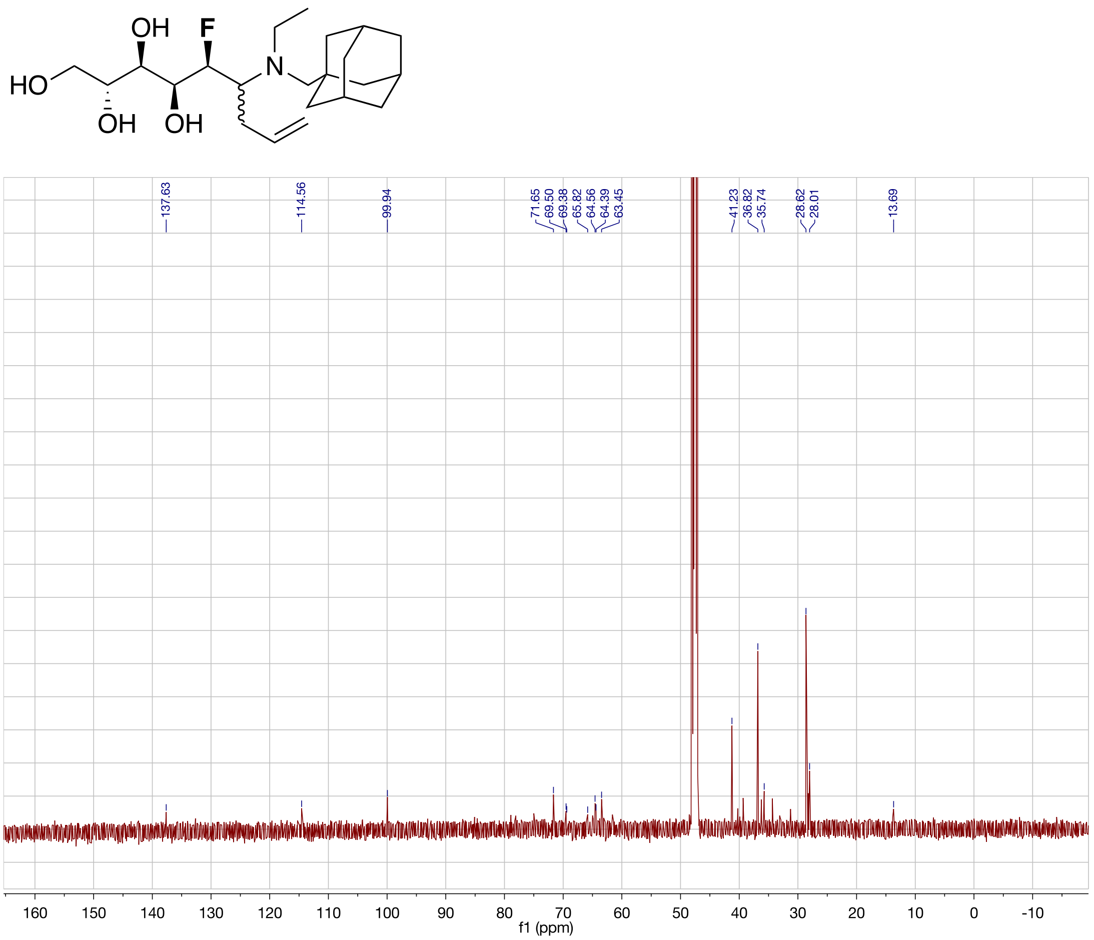

Figure S12. ^{13}C NMR spectra of [^{19}F]Ctrl-FAC-FDG-1.
